# Massive hemothorax caused by intercostal artery pseudoaneurysm:a case report

**DOI:** 10.1186/s13019-021-01548-1

**Published:** 2021-05-31

**Authors:** Caiyang Liu, Ran Ran, Xiaoliang Li, Gaohua Liu, Chuanxi Wang, Ji Li

**Affiliations:** 1 Department of Cardiothoracic Surgery, The First People’s Hospital of Neijiang, No. 1866, West Section of Hanan Avenue, Shizhong District, Neijiang, 641000 Sichuan China; 2 Breast Surgery Center of Sichuan Cancer Hospital, Chengdu, 610041 China

**Keywords:** Intercostal artery pseudoaneurysm, Massive hemothorax, Covered stent grafting, Surgical management,case report

## Abstract

**Background:**

Intercostal artery pseudoaneurysm is rare and at the risk of rupture. The aetiology is always reported to be iatrogenic and traumatic injury. Embolisation is the most common therapeutic method. Here, we report a case of spontaneous intercostal artery pseudoaneurysm and cured by combining covered stent grafting and surgical management.

**Case presentation:**

A 60-year-old man complained of acute right back pain for 5 h. Computed tomography showed right massive hemothorax and a giant mass with distinct feeding vessel originated from the thoracic aorta within the right hemithorax. Thoracocentesis was performed, and then a covered stent was positioned across the origin of the feeding vessel. The patient was diagnosed with intercostal artery pseudoaneurysm. Finally, we successfully resected the pseudoaneurysm and ligated the proximal part of the artery. Histologic examination have proved the diagnosis. The postoperative course was uneventful, and the patient was discharged on postoperative day 10. There is no recurrence reported during follow-up.

**Conclusions:**

Spontaneous intercostal artery pseudoaneurysm is extremly rare. Delayed hemothorax due to rupture of the pseudoaneurysm may occur years after the formation. Early diagnosis is important and a combined treatment of endovascular intervention and surgical management is feasible, especially for the case of ruptured large tumour-like mass presentation of the pseudoaneurysm.

## Background

Intercostal artery pseudoaneurysm is rare and has mostly been described through case reports [1–24]. Different from the true aneurysm, pseudoaneurysm is a hematoma contiguous with a defect in an arterial wall. It has the risk of rupture and sometimes may cause hemothorax [2, 3], as in our case, and can be potentially life-threatening. Most of them have been associated with surgical interventions or blunt thoracic trauma [6, 9]. Embolisation is the most common therapeutic method [2, 7, 10]. We report a case of spontaneous intercostal artery pseudoaneurysm and cured by combining covered stent grafting and surgical management.

## Case presentation

**Fig. 1:**
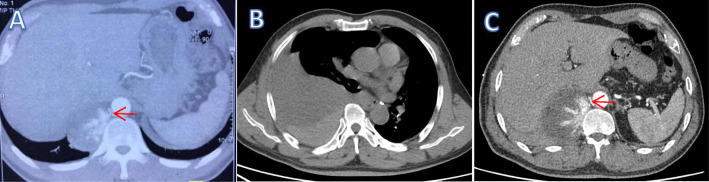
**a**. Chest CT 3 years prior showed a mass between the right spine and diaphragm. **b**. Chest CT showed right massive hemothorax. **c**. Chest CT showed growing mass with distinct feeding vessel

**Fig. 2:**
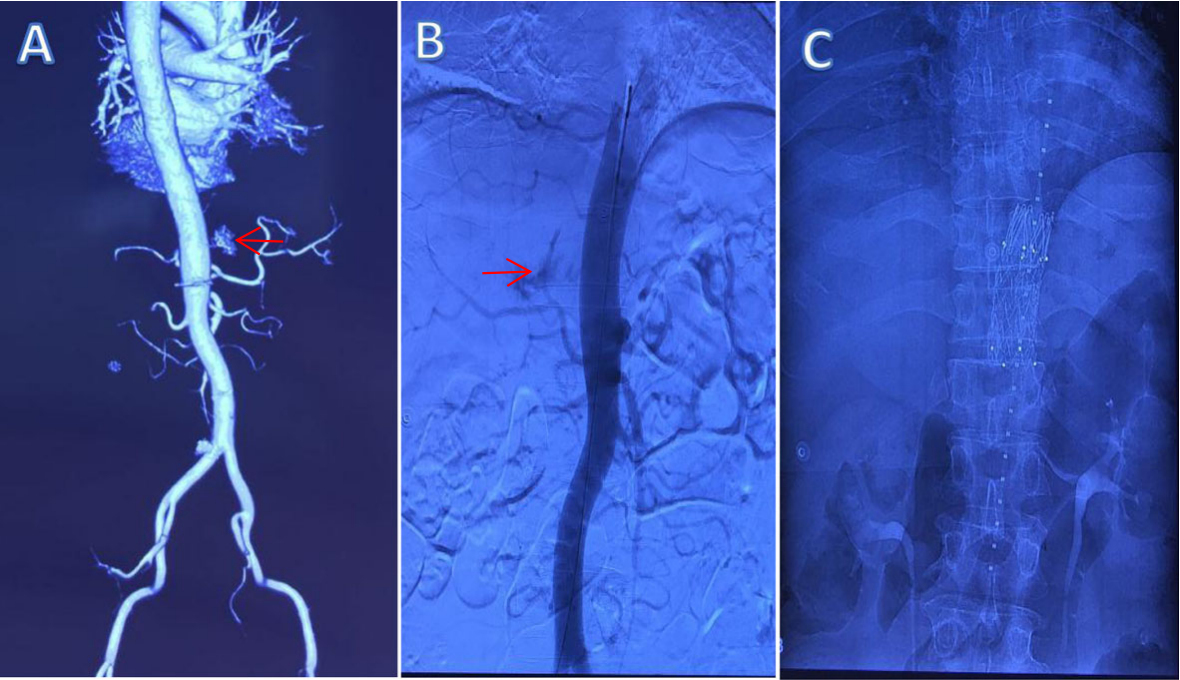
**a**. Three-dimensional reconstruction of the arterial vasculature. **b**. Arteriography showed a tortuous right eleventh intercostal artery with active bleeding in its distal area. **c**. A covered stent was positioned across the origin of the right eleventh intercostal artery

**Fig. 3:**
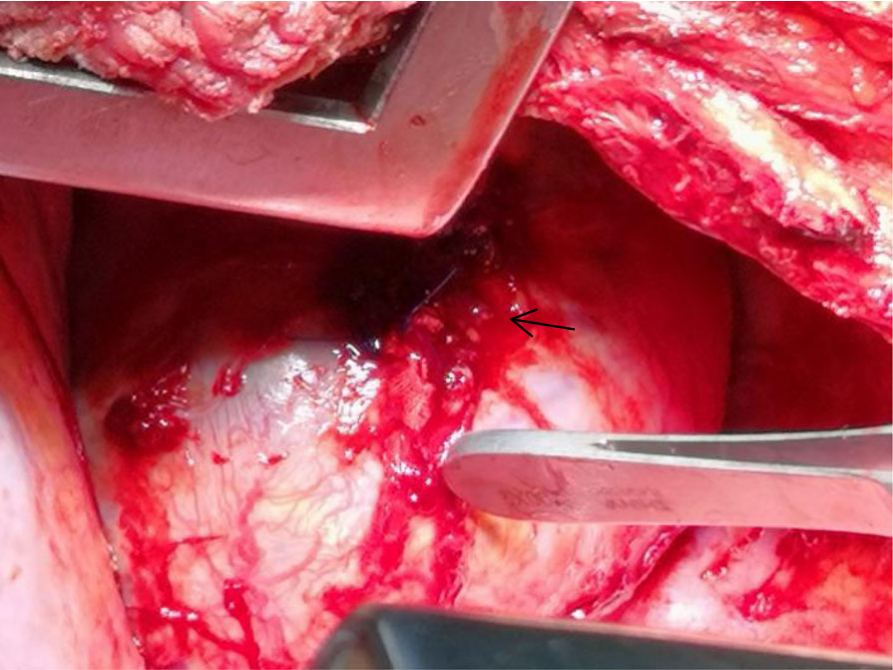
A giant pseudoaneurysm with huge surface tension attached to the right spine through a broad-based pedicle and there was still an active bleeder on its surface

A 60-year-old man was referred to our hospital by an ambulance because of acute right back pain for 5 h. The pain was constant and sharp. Firstly, it was limited to an area just inferior to the tip of his right scapula. But it subsequently radiated to his right chest. There was no associated fever, coughing, shortness of breath, or hemoptysis. He denied any falls or other trauma. He had a history of hypertension for about 4 years without standard treatment and highest reached 180/95mmhg. Chest computed tomography (CT) 3 years prior showed a mass between the right spine and diaphragm measuring 6.4 × 4.3 × 6.3 cm (Fig. 1a). But he did not undergo any kind of medical treatment until this admission. Body temperature was 36.4 °C, heart rate was 105 beats/min, blood pressure was 98/53mmhg, respiratory rate was 20 breaths/min, and oxygen saturation was 96%. Had pallor of conjunctivae. Right-side breath sounded weaken, left-side was normal. No moist or dry rales. No absence of heart murmurs or muffled heart tones. No midline spine or paraspinal tenderness. Laboratory examination revealed a serum low hemoglobin (74 g/l), high levels of serum D-dimer (6.08 mg/l) and fibrin degradation products (16.50 mg/l). CT showed right massive hemothorax and a 8.1 × 5.9 × 7.5 cm enhanced well-circumscribed mass within the right hemithorax (Fig. 1b). There was a distinct feeding vessel to this mass, which originated from the thoracic aorta (Fig. 1c). Three-dimensional reconstruction of the arterial vasculature suggested a right eleventh intercostal artery pseudoaneurysm (Fig. 2a). We diagnosed that the hemothorax was caused by rupture of the eleventh intercostal artery pseudoaneurysm. Thoracocentesis was performed, and 850 mL of bloody fluid was drained. The symptom of pain was obviously eased and the patient recovered from hemorrhagic shock with blood transfusion. But more than 200 ml/day of blood was drained after the thoracocentesis. On the third hospital day, an operation was planned because of increased hemothorax on chest radiographs and the progression of anemia. Given the large feeding artery emanating from the thoracic aorta, the patient underwent a covered stent grafting to minimize the risk of intraoperative bleeding before the operation. Arteriography showed a tortuous right eleventh intercostal artery with active bleeding in its distal area (Fig. 2b). An endurant II stent graft system ETFC2828C49EE was then positioned across the origin of the right eleventh intercostal artery (Fig. 2c). We subsequently performed a right posterolateral thoracotomy and a giant pseudoaneurysm with huge surface tension was identified. It attached to the right spine through a broad-based pedicle and there was still an active bleeder on its surface (Fig. 3). We found it contains substantial thrombus after an incision was made. The aneurysmectomy and ligation of the proximal part of the eleventh intercostal artery were successful. Intraoperative blood loss was 500 ml. Histologic examination showed that aneurysmal sac was consisted of fibrous tissue with no arterial structure, indicating that it was a pseudoaneurysm. The postoperative course was uneventful, and the patient was discharged on postoperative day 10. He was required to follow-up 1 month later and CT showed no recurrence.

## Discussion

Communicating with the flowing blood, pseudoaneurysm is a collection of blood outside the vessel lumen [25]. The absence of a 3-layered arterial wall differentiates it from a true aneurysm. Intercostal artery pseudoaneurysms are rare, as far as we know, there are 24 cases reported in the English literature [1–24]. The clinical findings of these cases are summarized in the Table 1. Ten patients were women and 14 were men. Their age ranged from 9 to 86 years (mean,55). Symptoms were hemothorax in 10 patients, pulsatile mass in 4 patients, hemoptysis in 2 patients, hematoma in 2 patients, acute chest or back pain in 3 patients, and hematemesis in 1 patient. The rest 2 patients without any symptoms were found accidentally by radiological examination. Aetiology of the cases described was iatrogenic in 17 patients, and traumatic in 6 patients. Cause of one patient without any medical history and chest trauma was unclear and,as well as this case, might be spontaneous.

**Table 1:** Reported cases of intercostal artery pseudoaneurysm

Study	Age (y)	Sex	Symptom	measuring (cm)	Diagnosis	Cause	Treatment	Ref
Wallace	24	M	Acute chest pain	8 × 12	CT	Aortic coarctation	Oversewing	[1]
Casas	85	M	Hemothorax	一	CT + AR	Percutaneous biliary procedure	Embolization	[2]
Atherton	32	F	Hemothorax	1.5	一	Thoracoscopic sympathectomy	Oversewing	[3]
Callaway	69	M	Pulsatile mass	2	DUS + AR	Sternotomy wiring	Covered stent grafting	[4]
Bluebond-Langner	59	M	Hematoma	3.5	CT + DUS	Retroperitoneal laparoscopic nephrectomy	Conservative management	[5]
Aoki	68	M	Hemothorax	1.5	CT	Blunt trauma	Aneurysmectomy plus proximal ligation (unsuccessful embolization)	[6]
Seishiro Sekino	36	M	Hemothorax	2 × 3	DUS + CT + AR	Stab wound	Embolization	[7]
Sebastian	71	M	Pulsatile mass	2.1	DUS	Median sternotomy	Thrombin injection	[8]
Kawai	59	F	Hemothorax	1	CT	Thoracoscopic lung resection	Aneurysmectomy plus proximal ligation	[9]
Melloni	70	F	Hematoma	一	CT + AR	Percutaneous lung biopsy	Embolization	[10]
Long	63	M	Hemothorax	0.8	CT + AR	Thoracentesis	Unsuccessful embolization	[11]
Guy Lenders	86	F	Pulsatile mass	3.2 × 1.7	CT + DUS	TAVI	Thrombin injection	[12]
Jourabchi	44	M	一	2 × 3	CT + AR	Transarterial Chemoembolization	Embolization	[13]
Romero	66	F	Hemothorax	一	CT + AR	Blunt trauma	Embolization	[14]
Chalapathi	32	M	Hematemesis	一	CT + AR	corrosive poisoning	Embolization	[15]
Vajtai	58	F	Pulsatile mass	2.5	DUS	Liver Biopsy	Thrombin injection	[16]
Takamure	49	F	Hemothorax	1.5 × 1	autopsy	Blunt trauma	一	[17]
Junck	63	M	Acute back pain	1.4 × 1.6	CT + AR	Spontaneous	Embolization	[18]
Agarwal	47	F	Acute back pain	一	CT + AR	Blunt trauma	Conservative management	[19]
Philippot	40	M	Hemoptysis	一	CT + AR	lobectomy	Embolization	[20]
Wu	9	F	Hemothorax	一	CT + AR	Thoracostomy	Embolization	[21]
Sharma	34	F	Hemoptysis	一	CT + AR	Pulmonary tuberculosis	Embolization	[22]
Casper	82	M	Hemothorax	一	DUS + CT + AR	Thoracentesis	Embolization	[23]
Rossi	82	M	Pulmonary mass	6 × 5.5	DUS + CT + AR	VATS	Embolization	[24]
This case	60	M	Hemothorax	8.1 × 7.5	CT + AR	Spontaneous	Covered stent grafting →Aneurysmectomy plus proximal ligation	

*CT* Computed tomography, *AR* arteriography, *DUS* Doppler ultrasound, *TAVI* Transaortic transcatheter aortic valve implantation

*VATS* video-assisted thoracoscopic surgery

Hemothorax was the most common presenting symptom in almost all the cases reported previously. So patients with massive hemothorax should be suspected to have an intercostal artery pseudoaneurysm, especially when they had chest trauma or underwent a surgical procedure via the intercostal space. The rupture of intercostal artery pseudoaneurysm causes brisk bleeding which may lead to shock or death. But, as reported, delayed hemothorax might occur two to 4 weeks after trauma or surgical procedures, so early diagnosis was possible and essential. Traditionally, diagnosis of an intercostal artery pseudoaneurysm was usually made by means of arteriography which allows endovascular treatment in a single procedure. But doppler ultrasound and CT are the two most primary diagnostic modality for intercostal artery pseudoaneurysm. In our case, intraoperative findings and pathology on resected tissues were also important in the diagnosis of intercostal artery pseudoaneurysm. Because, as for differential diagnosis, initially, we took pulmonary sequestration and mediastinal tumor into consideration.

The ruptured intercostal artery pseudoaneurysm was a good indication for endovascular intervention. Embolisation was considered to be the preferred treatment of a ruptured pseudoaneurysm. Many successful cases have been reported [7, 10, 13–15, 18, 21–24]. Generally speaking, microcoils were the first choice for embolisation [7, 13, 14, 18, 21, 24], but glue-lipiodol mixture [15], polyvinyl alcohol particles and gelfoam slurry [10, 22] could also serve as alternatives. Callaway et al. [4] reported a patient cured with covered stent. Conservative management [5, 19] and ultrasound-guided thrombin injection [8, 12, 16] had also been described. Only few cases involved open excision [1, 3, 6, 9]. Although endovascular intervention was often chosen, Sekino et al. [7] suggested that a pseudoaneurysm might have multiple blood supplies which sometimes lead to treatment failure. Actually, in our case, the covered stent did not interrupt the blood supply into the pseudoaneurysm completely. Furthermore, atelectasis occured because the thoracic cavity was filled with the hematoma that was difficult to absorb. Surgical removal of the gross hematoma should be performed to prevent infection and release the compressed lungs. So we formulated a two-step therapeutic schedule which was proved to be feasible.

## Conclusions

Intercostal artery pseudoaneurysms are rare and have a risk of rupture. Diagnosis before rupture is important. Embolisation is the most common therapeutic method because of its efficacy and low invasiveness, and surgical management is always reserved for embolisation failure. We report a patient with spontaneous intercostal artery pseudoaneurysm. Delayed hemothorax due to rupture of the pseudoaneurysm occurred more than 3 years after the formation. We emphasize the significance of combined treatment of endovascular intervention and surgical management, especially for the case of ruptured large tumour-like mass presentation of the pseudoaneurysm.

## Data Availability

Not applicable.

## Abbreviation

CT: Computed tomography

## References

[1] Wallace RB, Nast EP (1987). Postcoarctation mycotic intercostal arterial pseudoaneurysm [J]. Am J Cardiol.

[2] Darío Casas J, Perendreu J, Gallart A (1997). Intercostal artery pseudoaneurysm after a percutaneous biliary procedure: diagnosis with CT and treatment with transarterial embolization. J Comput Assist Tomogr.

[3] Atherton WG, Morgan WE (1997). False aneurysm of an intercostal artery after thoracoscopic sympathectomy.[J]. Ann R Coll Surg Engl.

[4] Callaway MP, Wilde P, Angelini G (2000). Treatment of a false aneurysm of an intercostal artery using a covered intracoronary stent-graft and a radial artery puncture.[J]. Br J Radiol.

[5] Bluebond-Langner R, Pinto PA, Kim FJ, et al. Recurrent bleeding from intercostal arterial pseudoaneurysm after retroperitoneal laparoscopic radical nephrectomy.[J]. Urology. 2003;60(6):1111–1. 10.1016/s0090-4295(02)01998-2 12475689

[6] Aoki T, Okada A, Tsuchida M, Hayashi J (2003). Ruptured intercostal artery pseudoaneurysm after blunt thoracic trauma.[J]. Thorac Cardiovasc Surg.

[7] Sekino S, Takagi H, Kubota H, Kato T, Matsuno Y, Umemoto T (2005). Intercostal artery pseudoaneurysm due to stab wound [J]. J Vasc Surg.

[8] Sebastian FA, Mendieta AC, Fernandez HA, et al. Post-sternotomy intercostal artery pseudoaneurysm. Sonographic diagnosis and thrombosis by ultrasound-guided percutaneous thrombin injection.[J]. Interact Cardiovasc Thorac Surg. 2009;9(4):722–4. 10.1510/icvts.2009.208116. 10.1510/icvts.2009.208116 19602496

[9] Kawai H, Ito M (2009). Intercostal artery pseudoaneurysm after thoracoscopic lung resection. Gen Thorac Cardiovasc Surg.

[10] Melloni G, Bandiera A, Crespi G, Zannini P (2012). Intercostal artery pseudoaneurysm after computed tomography-guided percutaneous fine needle aspiration lung biopsy. J Thorac Imaging.

[11] Long SS, Johnson PT, Fishman EK (2012). Intercostal artery Pseudoaneurysm due to thoracentesis: diagnosis with three-dimensional computed tomographic angiography [J]. J Comput Assist Tomogr.

[12] Guy L, Paul VS, Inez R (2012). Intercostal artery pseudoaneurysm: a rare complication of transaortic transcatheter aortic valve implantation. Interact Cardiovasc Thorac Surg.

[13] Jourabchi N, Sauk S, Hoffman C (2012). Intercostal Artery Pseudoaneurysm Formation after Irinotecan Transarterial Chemoembolization of a Spinal Metastasis from Colorectal Cancer. Case Rep Radiol.

[14] Romero DFG, Barrufet M, Lopezrueda A, et al. Ruptured intercostal artery pseudoaneurysm in a patient with blunt thoracic trauma: diagnosis and management. BMJ Case Reports. 2014, 2014;(1). 10.1136/bcr-2013-202019 PMC4078529 24966257

[15] Chalapathi Rao M, Rathi A, Reddy S, Sahu S (2014). Intercostal artery pseudoaneurysm complicating corrosive acid poisoning: diagnosis with CT and treatment with transarterial embolisation. Indian J Radiol Imaging.

[16] Vajtai Z, Roy N (2015). Intercostal artery pseudoaneurysm after ultrasound-guided liver biopsy: a case report and review of the literature. Ultrasound Quarterly.

[17] Takamure A , Nakagawa T , Kobayashi A , et al. Traumatic intercostal artery pseudoaneurysm following a bicycle accident. Forensic Sci Med Pathol. 2007;3(3):217. 10.1007/s12024-007-0019-0 25869167

[18] Emily J, Richard U, et al. Ruptured intercostal artery pseudoaneurysm: a rare cause of acute back pain. BMJ Case Rep. 2015. 10.1136/bcr-2015-209788 PMC4600782 26430227

[19] Agarwal A, Weerakkody Y, Marshall M (2017). Intercostal artery pseudoaneurysm with spontaneous resolution in the setting of an artery of Adamkiewicz. BMJ Case Rep.

[20] Philippot Q, Mahdjoub E, Carette MF, Fartoukh M, Voiriot G (2017). Intercostal artery Pseudoaneurysms. A rare cause of hemoptysis recurrence. Am J Respir Crit Care Med.

[21] Wu SH, Wu DK (2018). Active bleeding from intercostal artery pseudoaneurysm after a percutaneous tube thoracostomy drainage procedure: diagnosis with CT angiography and treatment with transarterial coil embolisation. BMJ Case Rep.

[22] Sharma M, Singhal M, Kamble R, et al. Intercostal artery pseudoaneurysm in pulmonary tuberculosis – A rare cause of hemoptysis: A case report with review of the literature. Lung India. 2019;36(1). 10.4103/lungindia.lungindia_87_18 PMC6330799 30604707

[23] Casper KP, Sanchirico PJ, Pfeiffer DC. Intercostal artery pseudoaneurysm following thoracentesis: Multi-modal imaging and treatment. BMC Med Imaging. 2019;19(1). 10.1186/s12880-019-0333-5 PMC6487039 31029094

[24] Rossi G, Perrillo M, Flora M, et al. Incidental diagnosis and therapeutic approach of an iatrogenic intra-parenchymal pulmonary intercostal artery pseudoaneurysm: a case report. Monaldi Arch Chest Dis. 2019;30, 89(3). 10.4081/monaldi.2019.1094 31505922

[25] Lee WK, Roche CJ, Duddalwar VA, Buckley AR, Morris DC (2001). Pseudoaneurysm of the uterine artery after abdominal hysterectomy: radiologic diagnosis and management. Am J Obstet Gynecol.

